# A preliminary checklist of the freshwater snails of Sabah (Malaysian Borneo) deposited in the BORNEENSIS collection, Universiti Malaysia Sabah

**DOI:** 10.3897/zookeys.673.12544

**Published:** 2017-05-15

**Authors:** Ting Hui Ng, Jasrul Dulipat, Junn Kitt Foon, Manuel Lopes-Lima, Alexandra Zieritz, Thor-Seng Liew

**Affiliations:** 1 Department of Biological Sciences, National University of Singapore, 14 Science Drive 4, Singapore 117543, Republic of Singapore; 2 Department of Biology, Faculty of Science, Chulalongkorn University, Phayathai Road, Pathumwan District, Bangkok 10330, Thailand; 3 Institute for Tropical Biology and Conservation, Universiti Malaysia Sabah. Jalan UMS, 88450 Kota Kinabalu, Sabah, Malaysia; 4 CIBIO/InBIO - Research Center in Biodiversity and Genetic Resources, Universidade do Porto, Campus Agrário de Vairão, Rua Padre Armando Quintas, 4485-661 Vairão, Portugal; 5 School of Environmental and Geographical Sciences, University of Nottingham Malaysia Campus, Jalan Broga, 43500 Semenyih, Malaysia

**Keywords:** Diversity, Kalimantan, Mollusca, non-marine gastropods, North Borneo, Sarawak

## Abstract

Sabah, a Malaysian state at the north-eastern tip of Borneo, is situated in one of the Earth’s biodiversity hotspots yet its freshwater gastropod diversity remains poorly known. An annotated checklist of the freshwater gastropods is presented, based on specimens deposited in the *BORNEENSIS* collection of the Institute for Tropical Biology and Conservation at Universiti Malaysia Sabah, Malaysia. A KMZ file is also provided, which acts as a repository of digital images and complete collection data of all examined material, so that it can be shared and adapted to facilitate future research.

## Introduction

Mollusca is the second most diverse animal phylum after Arthropoda, and nearly 4000 species of gastropods have been described from freshwater habitats alone (Strong et al. 2008). Freshwater gastropods achieve the highest diversity and endemism in tropical South-east Asia which includes the ancient lakes of Sulawesi (Indonesia) and Inle (Myanmar), as well as large river systems like the Mekong river basin (Strong et al. 2008). However, the total number of known species might represent only half of the species diversity, and many species remain to be discovered and described (Lydeard et al. 2004). In addition, although the IUCN conservation status has been assessed for only approximately 10% of these known species, freshwater gastropods account for 20% of recorded mollusc extinctions (Lydeard et al. 2004, Strong et al. 2008).

Although the number of extinct molluscs recorded in Asia is far less than other regions (Régnier et al. 2009), it may not reflect the reality that Asian malacofauna face a vast variety of threats (Köhler et al. 2012). Rather, this paradox is more likely due to the expertise and knowledge of freshwater gastropod being biased towards other regions (Bouchet 1997, Cuttelod et al. 2011, Johnson et al. 2013). Despite the high diversity of freshwater molluscs in South-east Asia, research involving freshwater snails has been mainly concentrated on the field of medical malacology, with most of the focus being on the zoonotic parasites hosted by these snails (e.g., Lim et al. 1976, TROPMED Medical Group 1986). As a result, little else is known about the molluscan hosts themselves. Seminal work done by van Benthem Jutting (e.g., 1956, 1959) and Brandt (1974) provided the most comprehensive insights into the diversity and distribution of freshwater molluscs in Indonesia, parts of Peninsular Malaysia, and Thailand. Decades later little progress has been made to update the information, where large knowledge gaps remain in the biodiversity, ecology and physiology of South-east Asian freshwater molluscs (Köhler et al. 2012).

Borneo, the third largest island in the world, is regarded as one of the Earth’s biodiversity hotspots because of its high species richness and endemism and its highly threatened habitats (Myers et al. 2000). The overall diversity of freshwater gastropods in Borneo, however, remains poorly known compared to the rest of South-east Asia. Previous faunistic studies recorded not more than 30 freshwater gastropod species from Borneo (Issel 1874, Bock 1881, Aldrich 1889, von Martens and Thiele 1908, Solem 1964, Hill et al. 1997, Köhler and Glaubrecht 2002, Shabdin 2010, Ng et al. 2015a). In contrast, more than 300 freshwater gastropod species have been recorded in Indochina (Thailand, Myanmar, Laos, Cambodia) (Brandt 1974, Köhler et al. 2012) and from areas much smaller than Borneo. For example, in Java, more than 60 species are known (Marwoto et al. 2011) and in Singapore, around 20 species have been recorded (Clements et al. 2006, Tan et al. 2012, Ng et al. 2014, 2015b, 2016a, 2016b, 2016c).

**Figure 1.**
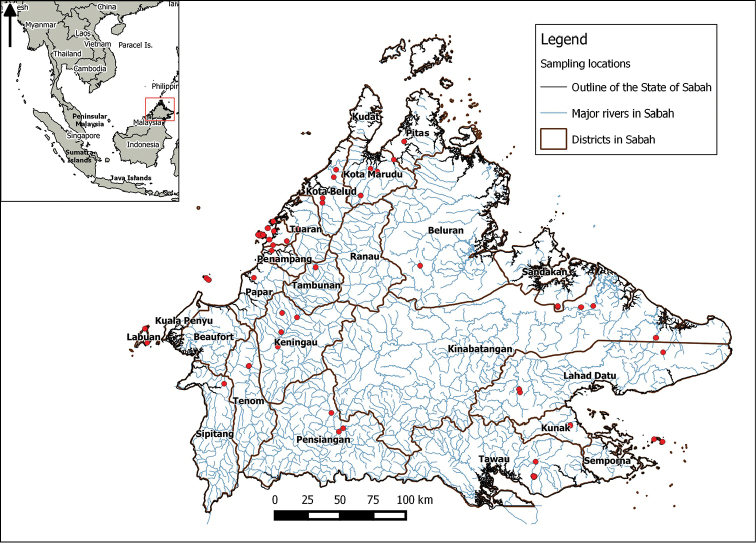
Map of Sabah with districts, major rivers and locations of the examined specimens (red dots). Inset shows the location of Sabah in South-east Asia.

In Borneo and particularly in the northern Malaysian state of Sabah (Figure 1), a complete species list that is based on primary data (i.e., based on accessible museum collections) does not exist. Previous studies of selected freshwater snails that focused on Sabah were limited to a few ecological (Supian and Ikhwanuddin 2002), parasitic (Lim et al. 1976), and pest control studies (Teo 2001, 2003, 2004). Presently, the task of identifying freshwater gastropods is complicated by the difficulty in obtaining comparative material from these past studies. Hence, an annotated checklist is provided for the freshwater gastropods in Sabah as a baseline framework and an identification tool for future studies. Instead of compiling species listed in previous studies or from unverifiable museum material, this checklist is based solely on the specimens collected from Sabah which are deposited in the *BORNEENSIS* collection of the Institute for Tropical Biology and Conservation at Universiti Malaysia Sabah, Malaysia.

All the specimens were catalogued using the *BORNEENSIS* Mollusca Collection in-house database, under the prefix of BOR/MOL #### to serve as stable specimen identifiers for future interrogation (e.g. Page 2016). In addition to the species list and representative images for each species, we also created a KMZ file as a repository of digital images and complete collection data of all the examined materials. All the specimen data are published under Creative Common license CC BY 4.0 so that it can be shared and adapted to facilitate future research (Meier and Dikow 2004, Miller et al. 2015). As the collection is based on a small number of surveys, this checklist is not a complete checklist for Sabah freshwater gastropods but it serves as a starting point to explore the diversity and taxonomy of freshwater gastropods not only in Sabah, but also for the rest of Borneo.

## Materials and methods

The freshwater gastropods deposited in the *BORNEENSIS* collection, Institute for Tropical Biology and Conservation, Universiti Malaysia Sabah, Malaysia (BOR/MOL), presently consists of 49 dry collection lots and 76 wet collection lots. A total of 849 specimens (between 1 and 59 specimens per lot) were examined. Approximately half of the collection lots were collected in 2016 by J. Dulipat, A. Zieritz, M. Lopes-Lima and T.S. Liew, whereas the rest were collected between 1997 and 2014. Most of the collections were made on the west coast of Sabah (Figure 1). The majority of the specimens were collected opportunistically and picked by hand and it is likely that smaller species were missed.

Identification was done to species level based on the shell morphology by referring to H. Adams (1874), A. Adams (1885), Smith (1894, 1895), von Martens and Thiele (1908), van Benthem Jutting (1956), Brandt (1974), and Tan et al. (2012). Synonymy follows Brandt (1974), Köhler and Glaubrecht (2001), Glaubrecht et al. (2009), Cowie (2015), and Eichhorst (2016). Distribution of each species in the checklist was summarised from the collection information, some of which contain words in the Malay language – *pulau*: island, *gua*: cave, *sungai*: river. The exact GPS coordinates for each collection lot was specified. If no exact GPS coordinates were available for a collection lot, approximate GPS coordinates were determined based on available locality information. Representative specimens of each of the collection lots were photograph using a single-lens reflex camera. Lastly, a KMZ file was created which consists of a main KML file and a supporting folder with all the photographs taken from each collection lot.

## Results and discussion

In total, 18 species were identified, from 14 genera and nine families of freshwater gastropods, including four non-native species. Their details are provided in the checklist below. The number of species recorded in this list is similar to that of Singapore (20 species), which has a much smaller landmass compared to Sabah (Clements et al. 2008, Ng et al. 2014, 2015, 2016b, 2016c). As such, it is clear that this list represents only a small fraction of the total freshwater gastropod diversity in Sabah which, together with bivalves, have been estimated to be 100 species (Solem 1964). Nevertheless, this checklist presents complete specimen information (Suppl. materials 1 and 2) for nearly two-thirds of previously known taxa in Borneo (Issel 1874, von Martens and Thiele 1908, Solem 1964, Hill et al. 1997, Shabdin 2010, Ng et al. 2015a).

## Systematics

### Family AMPULLARIIDAE Gray

#### Genus *Pila* Röding, 1798

##### Pila ampullacea

Taxon classificationAnimaliaArchitaenioglossaAmpullariidae

(Linnaeus, 1758)

Figure 2A

###### Synonyms.

*Helix ampullacea* Linnaeus, 1758; *Ampullaria sumatrensis* Philippi, 1851; *Ampullaria magnifica* Philippi, 1852; *Ampullaria turbinis* Lea, 1856; Ampullaria ampullacea var. javensis Nevill, 1885; Ampullaria turbinis var. subglobosa Nevill, 1885; Ampullaria turbinis var subampullacea Nevill, 1885; *Ampullaria dalyi* Blanford, 1903; Pachylabra turbinis race lacustris Annandale, 1920.

**Figure 2.**
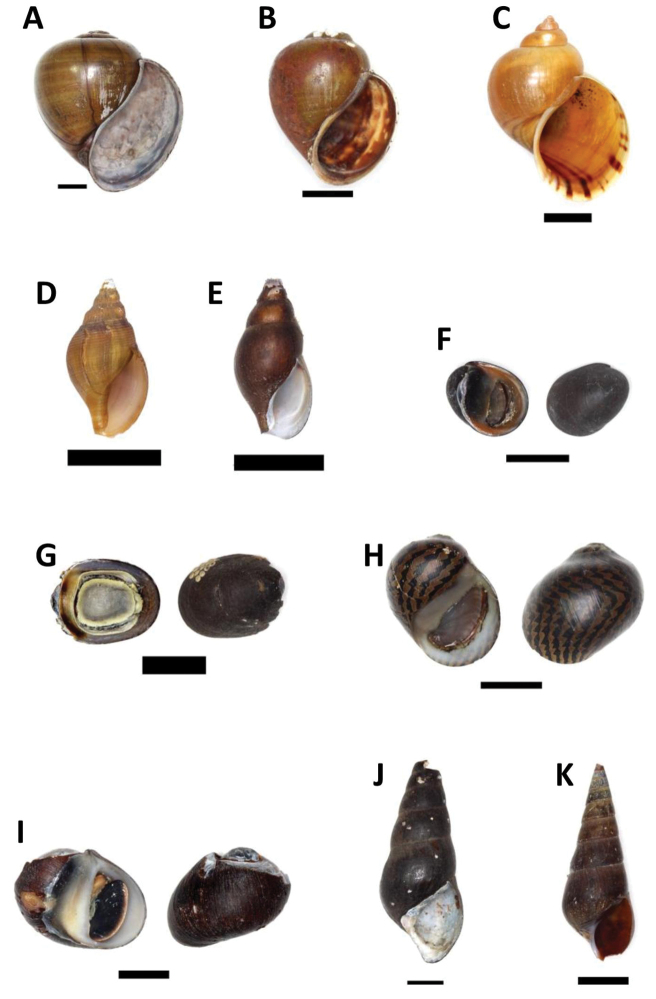
**A–C** Family Ampullariidae. **A** *Pila ampullacea* (Linnaeus, 1758) – BOR/MOL 8673 **B** *Pila scutata* (Mousson, 1848) – BOR/MOL 1758. **C** *Pomacea* sp. – BOR/MOL 1759 **D–E** Family Nassariidae **D** *Clea bangueyensis* EA Smith, 1895 – BOR/MOL 3397 **E** *Clea* sp. – BOR/MOL 8304 **F–I** Family Neritidae **F** *Neritina pulligera* (Linnaeus, 1767) – BOR/MOL 6705 **G** *Septaria porcellana* (Linnaeus, 1858) – BOR/MOL 8292 **H** *Vittina coromandeliana* (Sowerby, 1836) – BOR/MOL 8303. **I** *Vittina variegata* (Lesson, 1831) – BOR/MOL 6723 **J–K** Family Pachychilidae: *Sulcospira pageli* (Thiele, 1908) **J** BOR/MOL 1753 **K** BOR/MOL 3394. Scale bars 10 mm.

###### Material examined.

BOR/MOL3378, BOR/MOL3773, BOR/MOL3775, BOR/MOL8671, BOR/MOL8673, BOR/MOL8675, BOR/MOL8708.

###### Distribution and habitat.

Labuan, along the north-west coast of Sabah from Kota Belud, Kota Kinabalu, Penampang, to Papar, and in the interior town of Nabawan. Habitats include freshwater and mangrove swamps, ponds, and rivers.

###### Remarks.

This species was purchased on two separate occasions (BOR/MOL3773, BOR/MOL8708), ten years apart, from the same native market in Penampang.

##### Pila scutata

Taxon classificationAnimaliaArchitaenioglossaAmpullariidae

(Mousson, 1848)

Figure 2B

###### Synonyms.

*Ampullaria conica* W. Wood, 1828; *Ampullaria orientalis* Philippi, 1849; *Ampullaria borneensis* Philippi, 1852; *Ampullaria lubrica* Reeve, 1856; *Ampullaria vittata* Reeve, 1856; *Ampullaria complicata* Reeve, 1856; *Ampullaria stoliczkana* Nevill, 1877; *Ampullaria wellesleyensis* de Morgan, 1885; Pachylabra javanica var. fruhstorferi Kobelt, 1912; *Pachylabra* (*lubrica* var.?) *quadrasi* Kobelt, 1912.

###### Material examined.

BOR/MOL1758.

###### Distribution and habitat.

The single specimen was collected from a limestone hill in the Lower Kinabatangan valley.

###### Remarks.

The species has previously been recorded from Tuaran and appeared to have been eaten by local communities (Lim et al. 1976).

#### Genus *Pomacea* Perry, 1810

##### Pomacea

Taxon classificationAnimaliaArchitaenioglossaAmpullariidae

sp.

Figure 2C

###### Material examined.

BOR/MOL537, BOR/MOL1759, BOR/MOL8672, BOR/MOL8711.

###### Distribution and habitat.

Along the north-western coast from Kota Marudu to Kota Kinabalu and Tenom, and in the interior at Keningau. Habitats include freshwater swamps, rivers, and drains.

###### Remarks.

Ampullariids of the genus *Pomacea* originate from South America and are globally-invasive, causing widespread damage to paddy fields in South-east Asia (Joshi and Sebastian 2006). The harm brought about by *Pomacea* prompted various studies to control their spread, including in Sabah (Teo 2001, 2003, 2004). *Pomacea canaliculata* was first recorded from the state in 1992 (Yahaya et al. 2006), and has previously been found in paddy fields in Tuaran, Tambunan and Keningau (Teo 2004). Another species, the morphologically similar *Pomacea maculata*, has been widely introduced to South-east Asia (Hayes et al. 2008, 2012) and may also be established in Sabah. However, fresh materials were unavailable to confirm the identity of *Pomacea* from Sabah using molecular methods, which are the best for distinguishing between the species (Matsukura et al. 2013).

### Family NASSARIIDAE Iredale

#### Genus *Clea* H Adams & A Adams, 1855

##### Clea bangueyensis

Taxon classificationAnimaliaNeogastropodaNassariidae

EA Smith, 1895

Figure 2D

###### Material examined.

BOR/MOL552, BOR/MOL553, BOR/MOL554, BOR/MOL1762, BOR/MOL3105, BOR/MOL3385, BOR/MOL3396, BOR/MOL3397, BOR/MOL3595, BOR/MOL6719, BOR/MOL8676.

###### Distribution and habitat.

From northern to eastern Sabah: Kinabalu Park, Danum Valley, Tabin Wildlife Reserve, Kunak, and Tawau, and in the interior in Nabawan and Keningau. Collected from rivers and streams, some in the vicinity of limestone hills.

###### Remarks.

This species was first described from Pulau Banggi, off the north-east coast of Sabah.

##### Clea

Taxon classificationAnimaliaNeogastropodaNassariidae

sp.

Figure 2E

###### Material examined.

BOR/MOL8293, BOR/MOL8304, BOR/MOL8312, BOR/MOL8700.

###### Distribution and habitat.

Found from two localities close to Kota Kinabalu, rivers on the offshore island of Pulau Gaya, and Kiansom forest, approximately 20km from the city.

###### Remarks.

This species lacks the spiral striae of *Clea bangueyensis* and lacks the transverse striae of *Clea nigricans* A Adams, 1885, which was described from the neighbouring state of Sarawak (Adams 1885).

### Family NERITIDAE Rafinesque

#### Genus *Neritina* Lamarck, 1816

##### Neritina pulligera

Taxon classificationAnimaliaCycloneritimorphaNeritidae

(Linnaeus, 1767)

Figure 2F

###### Synonyms.

*Neritina rubella* Müller, 1774; *Neritina oculus* Röding, 1798; *Nerita rossmassleriana* Récluz, 1846; *Neritina larga* Hombron & Jacquinot, 1848; *Neritina brandti* Philippi, 1849; *Neritina pulligera subcanalis* Mousson, 1865; *Neritina subcanalis* Mousson, 1870; *Neritina sulcata* Tennison-Woods, 1878; *Neritina sumatrana* Dautzenberg, 1899.

###### Material examined.

BOR/MOL6705, BOR/MOL6713, BOR/MOL7929, BOR/MOL8294, BOR/MOL8298, BOR/MOL8301, BOR/MOL8308, BOR/MOL8311.

###### Distribution and habitat.

Labuan. The south-east in Tawau, and the north-west on Pulau Gaya and from Kota Kinabalu.

###### Remarks.

This species is generally found in clear, coastal freshwater streams and rivers from Okinawa, and south through South-east Asia and Australasia (van Benthem Jutting 1956, Brandt 1974, Eichhorst 2016).

#### Genus *Septaria* Férrusac, 1807

##### Septaria porcellana

Taxon classificationAnimaliaCycloneritimorphaNeritidae

(Linnaeus, 1858)

Figure 2G

###### Synonyms.

*Navicella aponogetonis* Vahl, 1795; *Sandalium pictum* Schumacher, 1817; *Navicella suborbicularis* Sowerby, 1825; *Navicella depressa* Lesson, 1831; *Navicella zebra* Lesson, 1931; *Navicella gaimardi* Récluz, 1841; *Navicella quoyi* Récluz, 1841; *Navicella affinis* Mousson, 1865; *Navicella fissa* Mousson, 1869; *Navicella haustrum* Reeve, 1856; *Navicella orbicularis* Reeve, 1856; *Navicella squamata* Dohrn, 1858; *Navicella pulcherrima* Tapparone-Canefri, 1883.

###### Material examined.

BOR/MOL8292, BOR/MOL8302, BOR/MOL8307.

###### Distribution and habitat.

All lots were collected from rivers of Pulau Gaya, off the coast of Kota Kinabalu.

###### Remarks.

This species is widespread in coastal freshwater streams, rivers and lakes from Sri Lanka to Australasia (van Benthem Jutting 1956, Eichhorst 2016).

#### Genus *Vittina* HB Baker, 1924

##### Vittina coromandeliana

Taxon classificationAnimaliaCycloneritimorphaNeritidae

(Sowerby, 1836)

Figure 2H

###### Synonyms.

*Nertina cochinsinae* Récluz, 1850; *Nerita ramosa* Meuschen, 1787; *Neritina paralella* Röding, 1798; *Neritina lugubris* Lamarck, 1822; *Neritina coromandeliana* Sowerby, 1836; *Neritina triangularis* Mörch, 1852; *Neritina pulcherrima* Mousson, 1857; *Neritina interstitialis* von Martens, 1877; *Neritina hieroglyphica* Wattlebled, 1886.

###### Material examined.

BOR/MOL8303.

###### Distribution and habitat.

Single lot collected from Kuari River on Pulau Gaya.

###### Remarks.

This species can be found in brackish estuarine areas (streams and mangrove swamps) from Japan through South-east Asia to Australasia, and India (Brandt 1974, Eichhorst 2016).

##### Vittina variegata

Taxon classificationAnimaliaCycloneritimorphaNeritidae

(Lesson, 1831)

Figure 2I

###### Synonyms.

*Neritina pulchra* Sowerby, 1836; *Neritina cuvieriana* Récluz, 1841; *Neritina turrita* Schmeltz, 1866; *Neritina granulosa* Schmeltz, 1869; *Nertina zelandicus* Mousson, 1869; *Neritella granulum* Schmeltz, 1974.

###### Material examined.

BOR/MOL6723.

###### Distribution and habitat.

Single lot collected from a river on Pulau Bohey Dulang, off the eastern town of Semporna.

###### Remarks.

This species can be found in coastal freshwater bodies in South-east Asia and Australasia, and the Pacific islands (van Benthem Jutting 1956, Brandt 1974, Eichhorst 2016).

### Family PACHYCHILIDAE P Fischer & Crosse

#### Genus *Sulcospira* Troschel, 1858

##### Sulcospira pageli

Taxon classificationAnimaliaSorbeoconchaPachychilidae

(Thiele, 1908)

Figure 2J,K

###### Synonym.

*Melania schmidti* Martens, 1908.

###### Material examined.

BOR/MOL542, BOR/MOL543, BOR/MOL544, BOR/MOL547, BOR/MOL548, BOR/MOL550, BOR/MOL1752, BOR/MOL1753, BOR/MOL3100, BOR/MOL3394, BOR/MOL3450, BOR/MOL3457, BOR/MOL3761, BOR/MOL3825, BOR/MOL5947, BOR/MOL5950, BOR/MOL6707, BOR/MOL6709, BOR/MOL6711, BOR/MOL6715, BOR/MOL6717, BOR/MOL6718, BOR/MOL6722, BOR/MOL8693, BOR/MOL8694, BOR/MOL8695, BOR/MOL8696, BOR/MOL8697, BOR/MOL8698, BOR/MOL8701.

###### Distribution and habitat.

West from Kota Marudu to Kota Kinabalu, in the interior in Tenom and Keningau, and eastern Sabah in Beluran, Kinabatangan, Lahad Datu, Kunak, Tawau. Found in forest streams and in the vicinity of limestone caves, rivers, and in streams along paddy fields.

###### Remarks.

This species has previously been synonymised with *Sulcospira schmidti* (Martens, 1908), which was also originally described from Borneo (Köhler and Glaubrecht 2001). Shells appear to be highly plastic, with material examined having shells with rounded or pointed basal lips, with or without raised ribs, and with or without spiral striae at the bottom of final body whorl. Other species of *Sulcospira* have previously been described from Borneo (see e.g., Köhler and Glaubrecht 2001, 2002, Köhler and Dames 2009), but pending further analysis and availability of fresh material for molecular sequencing, we tentatively consider all material conspecific.

### Family VIVIPARIDAE Gray

#### Genus *Sinotaia* Haas, 1839

##### Sinotaia guangdungensis

Taxon classificationAnimaliaArchitaenioglossaViviparidae

(Kobelt, 1906)

Figure 3A

###### Material examined.

BOR/MOL8674, BOR/MOL8709.

**Figure 3.**
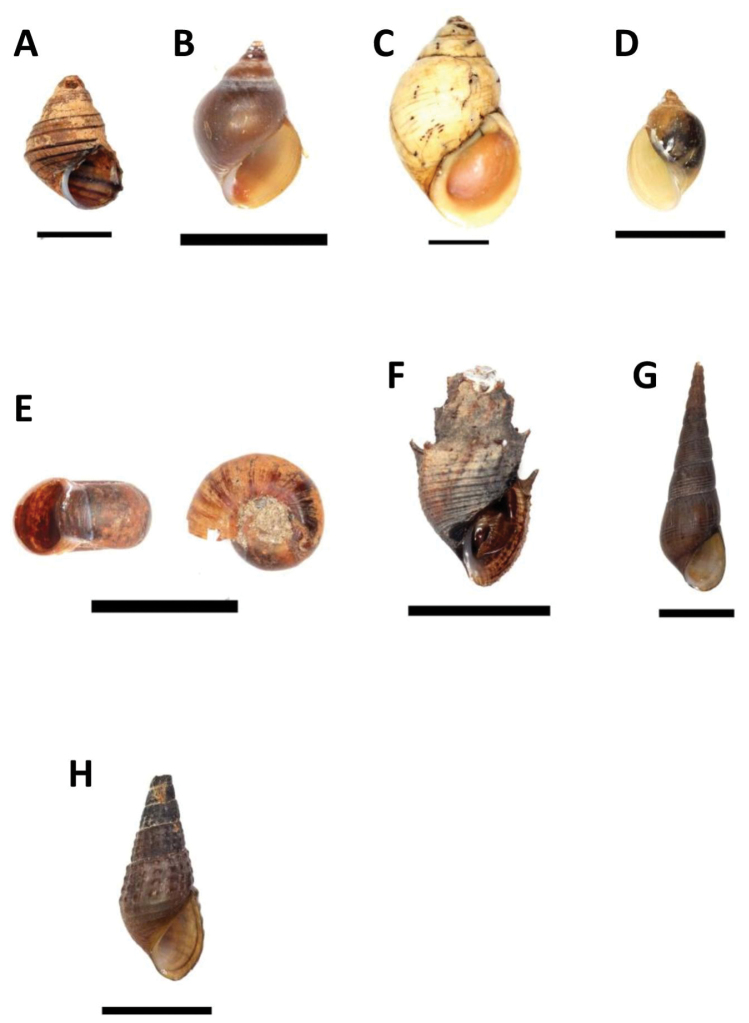
**A** Family Viviparidae: *Sinotaia guangdungensis* (Kobelt, 1906) – BOR/MOL 8709 **B–C** Family Paludomidae. **B** *Paludomus everetti* EA Smith, 1894 – BOR/MOL 3398 **C** *Paludomus luteus* Adams, 1874 – BOR/MOL 1225 **D** Family Physidae: *Physa acuta* Draparnaud, 1805 – BOR/MOL 3451 **E** Planorbidae *Indoplanorbis exustus* (Deshayes, 1834) – BOR/MOL 8681 **F–H** Family Thiaridae **F** *Mieniplotia scabra* (OF Müller, 1774) – BOR/MOL 8310 **G** *Melanoides tuberculata* (OF Müller, 1774) – BOR/MOL 6799 **H** *Tarebia granifera* (Lamarck, 1822) – BOR/MOL 8688. Scale bars 10 mm.

###### Distribution and habitat.

Collected from a paddy field stream in Kota Marudu, and from a pond in Nabawan.

###### Remarks.

*Sinotaia guangdungensis* is native to Southern China and has to date, been introduced to Peninsular Malaysia, Singapore, and Australia (Ng et al. 2014). This is a first record for Sabah.

### Family PALUDOMIDAE Stoliczka

#### Genus *Paludomus* Swainson, 1840

##### Paludomus everetti

Taxon classificationAnimaliaSorbeoconchaPaludomidae

EA Smith, 1894

Figure 3B

###### Material examined.

BOR/MOL545, BOR/MOL1226, BOR/MOL1227, BOR/MOL1754, BOR/MOL1755, BOR/MOL1756, BOR/MOL3127, BOR/MOL3398, BOR/MOL3596, BOR/MOL5853, BOR/MOL5870.

###### Distribution and habitat.

Eastern Sabah in Kunak, Danum Valley, Tawau, and Kinabatangan. Found in forest streams and in the vicinity of limestone caves.

###### Remarks.

Originally described from Batang Lupar in neighbouring Sarawak, and Gua Gomantong in the Kinabatangan area. The material in *BORNEENSIS* include shells collected from the vicinity of Gua Gomantong.

##### Paludomus luteus

Taxon classificationAnimaliaSorbeoconchaPaludomidae

Adams, 1874

Figure 3C

###### Material examined.

BOR/MOL1225.

###### Distribution and habitat.

Single lot found in the vicinity of Gua Gomantong in the Kinabatangan area.

###### Remarks.

Distinguished from *Paludomus everetti* by the lack of spiral striae at the suture.

### Family PHYSIDAE Fitzinger

#### Genus *Physa* Draparnaud, 1801

##### Physella acuta

Taxon classificationAnimaliaHygrophilaPhysidae

(Draparnaud, 1805)

Figure 3D

###### Material examined.

BOR/MOL3451.

###### Distribution and habitat.

Single lot collected from Kiansom forest, 20km inland from Kota Kinabalu.

###### Remarks.

This species is native to North America and has been widely introduced to neighbouring Sarawak and Brunei, on Borneo (Ali 1993, Ng et al. 2015b). This appears to be the first record of the species in Sabah.

### Family PLANORBIDAE Rafinesque

#### Genus *Indoplanorbis* Anandale & Prashad, 1920

##### Indoplanorbis exustus

Taxon classificationAnimaliaHygrophilaPlanorbidae

(Deshayes, 1834)

Figure 3E

###### Material examined.

BOR/MOL6716, BOR/MOL8681.

###### Distribution and habitat.

Collected from rivers in Tawau and Tuaran.

###### Remarks.

*Indoplanorbis exustus* has a wide distribution across Asia and is an intermediate host of zoonotic parasites (Liu et al., 2010). In Peninsular Malaysia, the species has been shown to host *Schistosoma spindale*, that causes cercarial dermatitis in infected humans (Chiew et al. 2009). The snail was not recorded from Tuaran in the 1970’s (Lim et al. 1976), and both lots in *BORNEENSIS* were only collected in 2016.

### Family THIARIDAE Gill

#### Genus *Mieniplotia* Low & Tan, 2014

##### Mieniplotia scabra

Taxon classificationAnimaliaNeotaenioglossaThiaridae

(OF Müller, 1774)

Figure 3F

###### Synonyms.

*Helix aspera* Gmelin, 1791; *Melania spinulosa* Lamarck, 1822; *Melania doreyana* Lesson, 1831; *Melania spinescens* Lesson, 1831; *Melanium granum* von dem Busch, 1842; *Melania scabrella* Mousson, 1848; *Melania acanthica* Lea, 1850; *Melania denticulata* Lea, 1850; *Melania pagoda* Lea, 1850; *Melania datura* Dohrn, 1858; *Melania elegans* Reeve, 1859; *Melania pugilis* Reeve, 1859; *Melania rugosa* Brot, 1860; *Melania snellemanni* Schepman, 1880; *Melania bockii* Brot, 1881; *Melania savinieri* Morlet, 1884; *Melania subcancellata* Boettger, 1890; *Melania pinguicola* Martens in Weber, 1897; *Melania varia* Bullen, 1904; *Melania intrepida* Fulton, 1914; *Melania sykesi* Degner, 1928.

###### Material examined.

BOR/MOL8300, BOR/MOL8306, BOR/MOL8310, BOR/MOL8690.

###### Distribution and habitat.

Off Kota Kinabalu on Pulau Gaya, and northern Sabah in Pitas. Collected from rivers.

###### Remarks.

This cryptogenic species is widespread across tropical Asia and is invasive around the world (Cianfanelli et al. 2016).

#### Genus *Melanoides* Olivier, 1804

##### Melanoides tuberculata

Taxon classificationAnimaliaNeotaenioglossaThiaridae

(OF Müller, 1774)

Figure 3G

###### Synonym.

*Melanoides fasciolata* Olivier, 1804.

###### Material examined.

BOR/MOL551, BOR/MOL1760, BOR/MOL1761, BOR/MOL6708, BOR/MOL6720, BOR/MOL6724, BOR/MOL6725, BOR/MOL6799, BOR/MOL7930, BOR/MOL7931, BOR/MOL8295, BOR/MOL8296, BOR/MOL8297, BOR/MOL8299, BOR/MOL8305, BOR/MOL8309, BOR/MOL8427, BOR/MOL8444, BOR/MOL8682, BOR/MOL8692, BOR/MOL8699.

###### Distribution and habitat.

Labuan. Widely-distributed throughout the state and on offshore islands, including Tenom, Kota Kinabalu, Pulau Gaya, Pulau Tiga, Tuaran, Kota Belud, Tawau, and Pulau Bohey Dulang and Pulau Bodgaya, off Semporna. Recorded from rivers, paddy fields, and concrete drains.

###### Remarks.

This species originates from West Asia and East Africa but has become widespread and invasive across the world (Pointier 1999).

#### Genus *Tarebia* H Ada& A Adams, 1884

##### Tarebia granifera

Taxon classificationAnimaliaNeotaenioglossaThiaridae

(Lamarck, 1822)

Figure 3H

###### Synonyms.

*Helix lineata* Gray in Wood, 1828; *Melania celebensis* Quoy & Gaimard, 1834; *Melania lirata* Benson, 1836; *Melania semigranosa* von dem Busch in Philippi, 1842; *Melania batana* Gould, 1843; *Melania coffea* Philippi, 1843; *Melania flavida* Dunker, 1844; *Melania verrucosa* Hinds, 1844; *Melania crenifera* Lea, 1850; *Melania lateritia* Lea, 1850; *Melania microstoma* Lea, 1850; *Melania rudis* Lea, 1850; *Melania granospira* Mousson, 1857; *Melania broti* Reeve, 1859; *Melania lyrata* Reeve, 1859; *Melania chocolatum* Brot, 1860; *Melania granospiralis* Zollinger, 1860; *Melania asperula* Brot, 1868; *Melania obliquigranosa* Smith, 1878; *Melania junghuhni* Martin, 1879; *Melania tjariangensis* Martin, 1905; *Melania kritjianensis* Martin, 1905; *Melania tjibodasensis* Leschke, 1914; *Melania margaritana* Leschke, 1914; *Melania martini* Oostingh, 1935.

###### Material examined.

BOR/MOL546, BOR/MOL3109, BOR/MOL3382, BOR/MOL6721, BOR/MOL8683, BOR/MOL8684, BOR/MOL8685, BOR/MOL8686, BOR/MOL8687, BOR/MOL8688, BOR/MOL8689, BOR/MOL8691.

###### Distribution and habitat.

Widely-distributed throughout the state, found from Nabawan, Keningau, Sepitang, Tuaran, Kota Belud, Pitas, Kota Marudu, Tawau. Most were collected from rivers.

###### Remarks.

This species is widespread in most water bodies from India to Australasia (van Benthem Jutting 1956).

## Supplementary Material

XML Treatment for Pila ampullacea

XML Treatment for Pila scutata

XML Treatment for Pomacea

XML Treatment for Clea bangueyensis

XML Treatment for Clea

XML Treatment for Neritina pulligera

XML Treatment for Septaria porcellana

XML Treatment for Vittina coromandeliana

XML Treatment for Vittina variegata

XML Treatment for Sulcospira pageli

XML Treatment for Sinotaia guangdungensis

XML Treatment for Paludomus everetti

XML Treatment for Paludomus luteus

XML Treatment for Physella acuta

XML Treatment for Indoplanorbis exustus

XML Treatment for Mieniplotia scabra

XML Treatment for Melanoides tuberculata

XML Treatment for Tarebia granifera

## Supplementary materials

Supplementary material 1 Collection data, image links and distribution data for freshwater snails of Sabah deposited in the BORNEENSIS collection, Universiti Malaysia Sabah Data type: KMZ file Explanation note: A KMZ file consisting of collection data, image links and distribution data. https://figshare.com/s/1d2e39c49f1e5eaa0840, https://doi.org/10.6084/m9.figshare.4725562.v1 This dataset is made available under the Open Database License (http://opendatacommons.org/licenses/odbl/1.0/). The Open Database License (ODbL) is a license agreement intended to allow users to freely share, modify, and use this Dataset while maintaining this same freedom for others, provided that the original source and author(s) are credited.Ting Hui Ng, Jasrul Dulipat, Junn Kitt Foon, Manuel Lopes-Lima, Alexandra Zieritz, Thor-Seng Liew

Supplementary material 2 A spreadsheet of collection and distribution data for freshwater snails of Sabah deposited in the BORNEENSIS collection, Universiti Malaysia Sabah Data type: Microsoft Excel spreadsheet Explanation note: A Microsoft Excel spreadsheet file consists of detailed collection and distribution data. https://figshare.com/s/1d2e39c49f1e5eaa0840, https://doi.org/10.6084/m9.figshare.4725562.v1 This dataset is made available under the Open Database License (http://opendatacommons.org/licenses/odbl/1.0/). The Open Database License (ODbL) is a license agreement intended to allow users to freely share, modify, and use this Dataset while maintaining this same freedom for others, provided that the original source and author(s) are credited.Ting Hui Ng, Jasrul Dulipat, Junn Kitt Foon, Manuel Lopes-Lima, Alexandra Zieritz, Thor-Seng Liew
